# R2-ISS分期在新诊断多发性骨髓瘤预后评估中的应用

**DOI:** 10.3760/cma.j.cn121090-20230810-00058

**Published:** 2024-02

**Authors:** 婕 严, 东明 周, 晓雁 邵, 勇 徐, 兵 陈

**Affiliations:** 1 南京医科大学鼓楼临床医学院血液科，南京 210008 Department of Hematology, Nanjing Drum Tower Hospital Clinical College of Nanjing Medical University, Nanjing 210008, China; 2 南京大学医学院附属鼓楼医院血液科，南京 210008 Department of Hematology, Nanjing Drum Tower Hospital, Affiliated Hospital of Medical School, Nanjing University, Nanjing 210008, China

**Keywords:** 多发性骨髓瘤, R2-ISS, 1q21扩增, 双打击, 预后, Multiple myeloma, Second Revision of the International Staging System, 1q gain/amplification, Double-hit, Prognosis

## Abstract

**目的:**

探讨R2-ISS（The Second Revision of the International Staging System）分期在新诊断多发性骨髓瘤（NDMM）患者中的预后价值。

**方法:**

收集自2012年12月至2022年3月在南京医科大学鼓楼临床医学院血液科就诊的326例以免疫调节药物和（或）蛋白酶体抑制剂为一线治疗方案的NDMM患者临床资料，采用Kaplan-Meier法进行生存分析，Log-rank检验比较组间差异，Cox比例风险回归模型进行多因素分析。

**结果:**

①326例NDMM患者中男性190例，中位年龄63岁，中位随访时间37个月。R2-ISS分期可进行有效的预后分层，特别是R-ISS Ⅱ期患者，R2-ISS Ⅰ期、Ⅱ期、Ⅲ期和Ⅳ期患者的中位无进展生存（PFS）期分别为52、29、20和15个月（*P*<0.001），中位总生存（OS）期分别为91、60、44和36个月（*P*<0.001）。多因素分析显示ISS Ⅱ期、ISS Ⅲ期、del（17p）、t（4；14）、1q+、LDH升高、年龄>65岁是影响OS的独立不良预后因素；ISS Ⅱ期、ISS Ⅲ期、del（17p） 、t（4；14）、1q+、LDH升高是影响PFS的独立不良预后因素。②R2-ISS分期C-index得分为0.724，优于R-ISS分期的0.678，预测效能更高。③R2-ISS Ⅲ期和Ⅳ期中含1q+在内的双打击患者中位 PFS期分别为20、15个月（*P*＝0.084），中位OS期为35、36个月（*P*＝0.786）。Ⅲ期中含1q+在内的双打击27例、1q+单一异常61例、不含1q+ 68例，三组的中位PFS期分别为20、18、21个月（*P*＝0.974），中位OS期分别为35、47、56个月（*P*＝0.042）。因此本研究将1q+赋值调整至1，重新分组后R2-ISS不同分期的中位PFS期和OS期差异均有统计学意义（*P*<0.001）。

**结论:**

R2-ISS分期预后分层价值优于R-ISS分期，特别是对异质性较强的R-ISS Ⅱ期人群，调整含1q+在内的双打击赋值后可进一步优化R2-ISS分期。

## Abstract (en)

**Objective:**

To investigate the prognostic value of the Second Revision of the International Staging System （R2-ISS） in patients with newly diagnosed multiple myeloma （NDMM）.

**Methods:**

The retrospective study was performed in 326 NDMM patients with immunomodulatory drugs and/or proteasome inhibitors as the first-line treatment attending the Department of Hematology, Nanjing Drum Tower Hospital Clinical College of Nanjing Medical University, Nanjing, China, from December 2012 to March 2022. The Kaplan-Meier method was used for the survival analysis, with the Log-rank test comparing the between-group differences and Cox proportional risk regression modeling A multifactorial analysis was performed.

**Results:**

①326 patients were included in the study, 190 of whom were males. The median age was 63 years, and the median followup time was 37 months. R2-ISS can effectively predict prognosis, particularly for R-ISS Ⅱ patients. The median progression-free survival （PFS） time of R2-ISS Ⅰ, R2-ISS Ⅱ, R2-ISS Ⅲ, and R2-ISS Ⅳ was 52, 29, 20, and 15 months （*P*<0.001）, while the median overall survival （OS） time was 91, 60, 44, and 36 months （*P*<0.001）. Multifactor analysis revealed that ISS Ⅱ, ISS Ⅲ, del（17p）, t（4;14）, 1q+, LDH increased, and age >65 years old were independent negative prognostic factors for OS. ISS Ⅱ, ISS Ⅲ, del（17p）, t（4;14）, 1q+, and LDH were independent negative prognostic factors for PFS. ②The C-index score of R2-ISS was 0.724, higher than that of R-ISS （0.678）, indicating high prediction efficiency. ③The median PFS for 1q+-related double-hit in R2-ISS Ⅲ and Ⅳ were 20, 15 months（*P*＝0.084） and the median OS were 35, 36 months（*P*＝0.786）, respectively. In R2-ISS Ⅲ, there were twenty-seven cases of 1q+-related double-hit, sixty-one cases of 1q+ single abnormality, and sixty-eight cases with no 1q+. The median PFS for the three groups were 20, 18, and 21 months （*P*＝0.974）, while the median OS was 35, 47, and 56 months （*P*＝0.042）, respectively. Adjusting the assignment of 1q+ to 1, the median PFS and OS of different R2-ISS stages differed significantly after regrouping （*P*<0.001）.

**Conclusion:**

The prognostic stratification value of R2-ISS is higher than R-ISS, particularly in the highly heterogeneous R-ISS Ⅱ population. Adjusting the assignment of the 1q+-related double-hit can improve R2-ISS, which should be validated in future studies with multi-center and expanded cases.

多发性骨髓瘤（MM）是一种异质性较强的浆细胞恶性增殖性疾病，约占血液系统肿瘤的10％，目前仍然不可治愈，总生存（OS）期差异较大，从数月到十余年不等[1]。尽管近年来新药的联合应用显著延长了MM患者的OS期和无进展生存（PFS）期，但仍有部分中高危患者未能从标准方案中获益，因此，初诊时精准的危险分层及预后评估对于MM患者实现个体化治疗、改善预后至关重要。

2022年欧洲骨髓瘤网络（EMN）在R-ISS（Revision of the International Staging System）分期基础上提出了新的分期系统R2-ISS（the Second Revision of the International Staging System）分期[2]，能够更好地进行危险分层，评估预后，特别是对大量R-ISS Ⅱ期患者。由于该分期系统是基于大量临床试验数据开发并验证的，目前尚未达成共识，仍需在临床实践中进行验证，因此，本研究回顾性分析我中心连续收治的新诊断多发性骨髓瘤（NDMM）患者的临床数据，分析R2-ISS分期下MM患者的OS和PFS，探讨1q+对MM患者预后的影响，现报道如下。

## 病例与方法

一、病例

回顾性分析自2012年12月至2022年3月在南京医科大学鼓楼临床医学院血液科连续收治的346例NDMM患者的基线特征及预后。所有患者均按照2015年IMWG诊断标准[3]进行诊断，并接受免疫调节药物（IMiD）（来那度胺或沙利度胺）和（或）蛋白酶体抑制剂（PI）（硼替佐米）作为一线治疗方案。其中326例（94.2％）患者可查询到以下临床数据并纳入研究，包括ISS分期、R-ISS分期、LDH、高危细胞遗传学特征［1q+、del（17p）、t（4；14）］，且随访时间均超过1年。本研究遵循的程序符合《世界医学协会赫尔辛基宣言》要求，并已获得我院伦理委员会批准（批件号：2023-043-02）。

二、研究方法

1. FISH检查：根据EMN关于规范MM荧光原位杂交技术的研讨会，对每个样本至少选取200个骨髓瘤细胞核进行检测，并定义了阳性结果[4]。应用FISH检测CD138^+^骨髓浆细胞染色体区域1q21、17p13、13q14.3、t（11；14）（q13；q32）、t（6；14）（p21；q32）、t（4；14）（p16；q32）和t（14；16）（q32；q23）的细胞遗传学异常。如果样本中≥20％的细胞核至少有三个1q拷贝，则定义为1q+阳性；如果样本中≥20％的细胞核检出17p13、13q14.3，则定义为del（17p）和del（13q）；如果样本中≥10％的细胞核检出t（11；14）（q13；q32）、t（6；14）（p21；q32）、t（4；14）（p16；q32）和t（14；16）（q32；q23），则定义为t（11；14）、t（4；14）、t（6；14）和t（14；16）。

2. R2-ISS分期：根据各基线危险因素进行赋值：ISS Ⅱ期赋1分、ISS Ⅲ期赋1.5分、del（17p）赋1分、LDH升高赋1分、t（4；14）赋1分，1q+赋0.5分，将MM患者分别定义为低危（Ⅰ期，0分）、低中危（Ⅱ期，0.5～1分）、中高危（Ⅲ期，1.5～2.5分）和高危（Ⅳ期，3～5分）[2]。

3. 含1q+在内的双打击及三打击：本研究根据Schmidt等[5]提出的“双打击”效应，将1q+合并t（4；14）、1q+合并del（17p）、1q+合并t（14；16）定义为含1q+在内的双打击，1q+合并t（4；14）及del（17p）、1q+合并t（14；16）及del（17p）、1q+合并t（4；14）及t（14；16）均定义为含1q+在内的三打击。由于没有关于1q21具有3个（gain）或≥4个（amp）拷贝的细胞核数量的数据，因此无论获得区域的拷贝数如何，本研究将gain/amp（1q21）归为一组，并用符号1q+表示。

三、随访

通过查阅住院、门诊病历和电话随访获得所有患者资料。随访截至2023年3月31日，中位随访时间37个月。

四、预后评价

参照IMWG 2006疗效评价标准。PFS期定义为从开始诱导治疗至疾病进展、复发、死亡或终止随访的时间。OS期定义为从疾病确诊至因任何原因死亡或终随访的时间。

五、统计学处理

本研究采用SPSS 25.0软件进行数据分析。分类变量采用χ^2^检验或Fisher精确概率检验。采用Kaplan-Meier法进行生存分析并绘制生存曲线和单因素分析，Log-rank检验比较组间差异，寿命表法估算生存率。多因素分析采用Cox比例危险回归模型。使用一致性指数（C-index）评价不同预后模型的预测效能，1.0提示最佳预测效能，0.5则提示完全无预测效能。所有检验均为双侧，以*P*<0.05为差异有统计学意义。

## 结果

一、临床特征

查阅本中心346例NDMM患者的临床资料，其中326例患者R2-ISS分层变量完整，故而纳入本研究。纳入评估与未纳入评估患者在年龄、性别、骨髓瘤分型、ISS分期、自体造血干细胞移植（auto-HSCT）状态、治疗方案的分布上差异无统计学意义。被评估的326例患者中男性190例（58.2％），中位年龄为63岁，中位随访时间为37个月。71例（21.8％）为R-ISS Ⅲ期，15例（4.6％）合并淀粉样变性，29例（8.8％）合并髓外浆细胞瘤，3年PFS率28％，5年OS率31％。140例（42.9％）患者接受了PI联合IMiD方案治疗，诱导治疗后94例（28.8％）患者接受了auto-HSCT，治疗方案在疾病各期之间的分布差异无统计学意义（*P*＝0.840）（表1）。18例（5.5％）二线治疗方案使用了达雷妥尤单抗，9例（2.8％）复发后接受了CAR-T细胞治疗。本中心与EMN队列的可评估训练集患者相比，男性、LDH升高、del（17p）和t（4；14）的患者比例等基线数据差异无统计学意义，而年龄、ISS分期、R-ISS 分期、1q+、一线治疗方案、auto-HSCT比例差异则有统计学意义（*P*<0.05）（表2）。

**表1:** 326例新诊断多发性骨髓瘤患者在R2-ISS分期中的分布[例（%）]

因素	R20（0）ISS Ⅰ期（43例）	R20（0）ISS Ⅱ期（89例）	R20（0）ISS Ⅲ期（158例）	R20（0）ISS Ⅳ期（36例）
治疗方案				
IMiD	5（12）	10（11）	19（12）	8（22）
IMiD0（0）PI	19（44）	41（46）	67（42）	13（36）
PI	19（44）	38（43）	72（46）	15（42）
自体造血干细胞移植	16（37）	25（28）	45（28）	8（22）
R0（0）ISS分期				
Ⅰ期	43（100）	13（15）	0（0）	0（0）
Ⅱ期	0（0）	76（85）	111（70）	12（33）
Ⅲ期	0（0）	0（0）	47（30）	24（67）
无危险因素	43（100）	0（0）	0（0）	0（0）
ISS分期				
Ⅰ期	43（100）	26（29）	5（3）	0（0）
Ⅱ期	0（0）	63（71）	69（44）	5（14）
Ⅲ期	0（0）	0（0）	84（53）	31（86）
LDH				
升高	0（0）	3（3）	25（16）	26（72）
正常	43（100）	86（97）	133（84）	10（28）
del（17p）				
阳性	0（0）	0（0）	26（16）	22（61）
阴性	43（100）	89（100）	132（84）	14（39）
t（4；14）				
阳性	0（0）	3（3）	26（16）	4（11）
阴性	43（100）	86（97）	132（94）	32（89）
1q+				
阳性	0（0）	19（21）	90（57）	34（94）
阴性	43（100）	70（79）	68（43）	2（6）
1q+单一异常及双打击、三打击				
1q+单一异常	0（0）	18（20）	61（39）	13（36）
1q+合并t（4；14）	0（0）	0（0）	15（10）	1（3）
1q+合并del（17p）	0（0）	0（0）	12（7）	18（50）
1q+合并t（14；16）	0（0）	1（1）	0（0）	0（0）
t（4；14）合并del（17p）	0（0）	0（0）	0（0）	1（3）
含1q+的三打击	0（0）	0（0）	2（1）	2（5）

注 IMiD：免疫抑制剂；PI：蛋白酶体抑制剂；1q+：1q增益/扩增；ISS分期：国际分期系统；R-ISS：修订后的国际分期系统；R2-ISS：第二次修订的国际分期系统

**表2:** 本中心与欧洲骨髓瘤网络（EMN）队列的可评估训练集患者的基线特征和治疗方法比较[例（%）]

基线特征	本中心（326例）	EMN队列的可评估训练集（2 226例）	*P*值
中位年龄[岁，*M*（范围）]	63（43～88）	60（54～65）	
>65岁	127（39）	506（23）	<0.001
男性	190（58）	1 271（57）	0.686
ISS分期			<0.001
Ⅰ期	74（23）	830（37）	
Ⅱ期	137（42）	845（38）	
Ⅲ期	115（35）	551（25）	
R-ISS分期			<0.001
Ⅰ期	56（17）	597（27）	
Ⅱ期	199（61）	1 372（62）	
Ⅲ期	71（22）	257（11）	
LDH升高	54（17）	363（16）	0.907
del（17p）	48（15）	258（12）	0.104
t（4；14）	33（10）	277（12）	0.231
1q+	143（44）	820（37）	0.014
一线治疗方案			<0.001
IMiD	42（13）	506（23）	
IMiD-PI	140（43）	1 485（67）	
PI	144（44）	235（11）	
自体造血干细胞移植	94（29）	1 855（83）	<0.001
R-ISS Ⅱ期（根据R2-ISS分期）	199（61）	1 372（62）	0.114
R2-ISS Ⅱ期	76（23）	517（23）	
R2-ISS Ⅲ期	111（34）	811（36）	
R2-ISS Ⅳ期	12（4）	44（3）	

注 1q+：1q 增益/扩增；IMiD：免疫抑制剂；PI：蛋白酶体抑制剂；ISS分期：国际分期系统；R-ISS：修订后的国际分期系统；R2-ISS：第二次修订的国际分期系统

二、R2-ISS分期的预后分层价值及预测效能

根据R2-ISS分期进行危险分层，326例NDMM患者被划分为Ⅰ期43例（13.1％），Ⅱ期89例（27.3％），Ⅲ期158例（48.4％），Ⅳ期36例（11.0％），中位PFS期分别为52、29、20、15个月（*P*<0.001），中位OS期分别为91、60、44、36个月（*P*<0.001）（图1）。

**图1:**
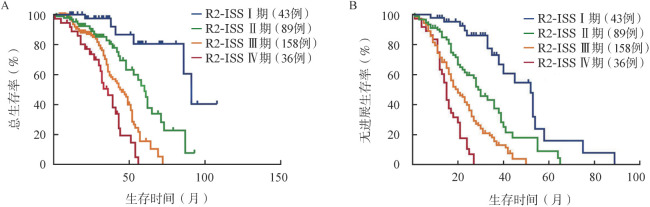
根据R2-ISS分期的新诊断多发性骨髓瘤患者的总生存（A）和无进展生存（B）曲线

R2-ISS分期可更好地区分R-ISS Ⅱ期中不良预后的患者，199例（61.0％）R-ISS Ⅱ期患者可再分为R2-ISS Ⅱ期76例（23.3％），Ⅲ期111例（34.0％），Ⅳ期12例（3.7％），与EMN队列的可评估训练集差异无统计学意义（表2）。三组中位PFS期分别为30、20、16个月（*P*<0.001），中位OS期分别为61、47、39个月（*P*<0.001）（图2）。

**图2:**
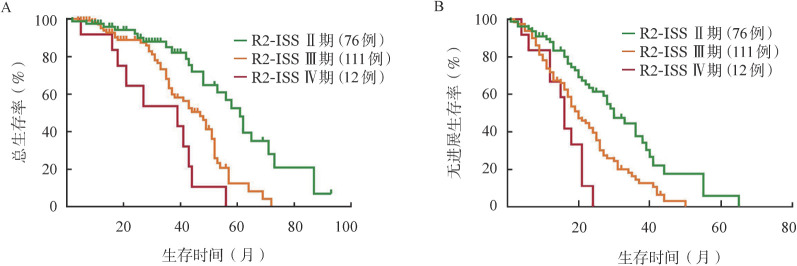
根据R2-ISS分期的R-ISS Ⅱ期新诊断多发性骨髓瘤患者的总生存（A）和无进展生存（B）曲线

采用C-index进行预后效能评价，R-ISS分期得分为0.678，R2-ISS分期得分为0.724，证实R2-ISS分期预后预测效能优于R-ISS分期，预后分层价值更优越。

三、不同危险因素的预后价值

通过Cox回归分析R2-ISS分期中的各个基线危险因素对患者OS及PFS的影响。单因素分析显示ISS Ⅱ期、ISS Ⅲ期、del（17p）、LDH升高、t（4；14）、1q+、年龄>65岁及是否行auto-HSCT是影响患者OS及PFS的预后因素（表3）。auto-HSCT可显著延长MM患者的OS期和PFS期（*P*<0.001）。将上述危险因素纳入多因素回归分析，结果显示，ISS Ⅱ期、ISS Ⅲ期、del（17p）、t（4；14）、1q+、LDH升高、年龄>65岁是影响OS的独立危险因素。ISS Ⅱ期、ISS Ⅲ期、del（17p）、t（4；14）、1q+、LDH升高是影响PFS的独立危险因素（表4）。

**表3:** R2-ISS分期中的危险因素对新诊断多发性骨髓瘤患者生存影响的单因素Cox回归分析

危险因素	PFS单因素分析		OS单因素分析	
	*HR* （95% *CI*）	*P*值	*HR* （95% *CI*）	*P*值
ISS Ⅰ期（参考值）	1			
ISS Ⅱ期	1.966（1.340～2.884）	0.001	2.953（1.723～5.064）	<0.001
ISS Ⅲ期	3.034（2.029～4.537）	<0.001	4.247（2.397～7.527）	<0.001
del（17p）	2.288（1.617～3.237）	<0.001	2.669（1.762～4.041）	<0.001
t（4；14）	1.791（1.184～2.711）	0.006	2.452（1.494～4.024）	<0.001
1q+	2.152（1.628～2.846）	<0.001	2.468（1.705～3.573）	<0.001
LDH	3.078（2.201～4.304）	<0.001	2.430（1.656～3.566）	<0.001
年龄>65岁	1.555（1.180～2.050）	0.002	2.591（1.813～3.703）	<0.001
IMiD（参考值）	1		1	
PI	1.074（0.737～1.565）	0.710	1.320（0.844～2.064）	0.224
IMiD-PI	0.820（0.558～1.205）	0.312	0.994（0.626～1.577）	0.979
自体造血干细胞移植	0.642（0.467～0.882）	0.006	0.474（0.291～0.773）	0.003

注 R2-ISS：第二次修订的国际分期系统；OS：总生存；PFS：无进展生存；*HR*：风险比；ISS：国际分期系统；1q+：1q 增益/扩增；IMiD：免疫抑制剂；PI：蛋白酶体抑制剂

**表4:** R2-ISS分期中的危险因素对新诊断多发性骨髓瘤患者生存影响的多因素Cox回归分析

危险因素	PFS多因素分析		OS多因素分析	
	*HR* （95% *CI*）	*P*值	*HR* （95% *CI*）	*P*值
ISS Ⅱ期	1.495（1.005～2.224）	0.047	1.834（1.040～3.234）	0.036
ISS Ⅲ期	1.880（1.215～2.909）	0.005	2.368（1.275～4.395）	0.006
del（17p）	1.526（1.044～2.231）	0.029	1.977（1.249～3.129）	0.004
t（4；14）	1.863（1.208～2.873）	0.005	2.831（1.641～4.884）	<0.001
1q+	1.504（1.101～2.054）	0.010	1.858（1.225～2.819）	0.004
LDH	2.336（1.615～3.380）	<0.001	1.821（1.181～2.807）	0.007
年龄>65岁	1.317（0.971～1.785）	0.076	2.529（1.713～3.736）	<0.001
自体造血干细胞移植	0.785（0.552～1.116）	0.178	0.805（0.473～1.370）	0.424

注 R2-ISS：第二次修订的国际分期系统；OS：总生存；PFS：无进展生存；ISS：国际分期系统； 1q+：1q 增益/扩增

四、1q+对NDMM OS和PFS的影响及重新赋值后的再分层价值

根据是否伴1q+进行分组，1q+ 143例（43.8％），不伴1q+ 183例（58.1％），两组中位PFS期分别为18、33个月（*P*<0.001），中位OS期分别为41、62个月（*P*<0.001）（图3）。

**图3:**
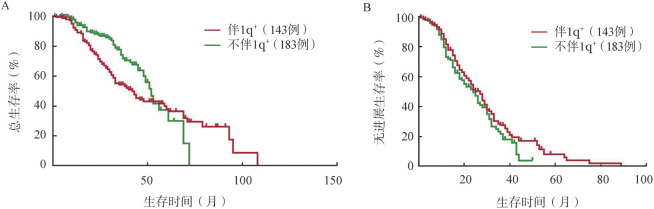
根据是否伴1q+分组的新诊断多发性骨髓瘤患者总生存（A）和无进展生存（B）曲线

1. R2-ISS Ⅲ期、Ⅳ期中含1q+在内双打击患者分别有27、19例，两组中位PFS期分别为20、15个月（*P*＝0.084），中位OS期分别为35、36个月（*P*＝0.786），差异均无统计学意义。表明R2-ISS Ⅲ期、Ⅳ期中存在预后相同的人群。

2. R2-ISS Ⅲ期中1q+在内的双打击、1q+单一异常、不含1q+三组的中位PFS期分别为20、18、21个月（*P*＝0.974），中位OS期分别为35、47、56个月（*P*＝0.042）（三打击因例数较少暂不纳入分析）。表明R2-ISS Ⅲ期包含着预后不同的患者。

3. Cox回归分析显示含1q+在内的双打击与不含1q+患者的OS风险比为2.025（*P*＝0.026），1q+单一异常与不含1q+患者的OS风险比为1.099（*P*＝0.753）。根据风险比将双打击中1q+赋值从0.5调整至1，对应1q+在内的双打击或三打击赋值则调整为2或3。R2-ISS Ⅲ期中26例双打击、2例三打击患者可划分至R2-ISS Ⅳ期。所有纳入评估患者重新划分为Ⅰ期43例（13.1％），Ⅱ期89例（27.3％），Ⅲ期130例（39.8％），Ⅳ期64例（19.6％），中位PFS期分别为52、29、20、16个月（*P*<0.001），中位OS期分别为91、60、49、35个月（*P*<0.001）（图4）。重新分组后的R2-ISS Ⅲ期主要包含61例1q+单一异常、68例无1q+，其中位PFS期分别为18、21个月（*P*＝0.870），中位OS期分别为47、56个月（*P*＝0.768），提示重新分组后的R2-ISS Ⅲ期患者预后差异无统计学意义。

**图4:**
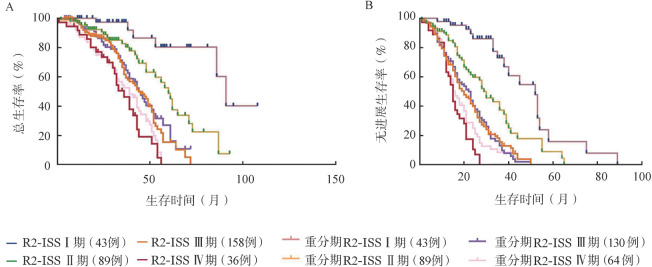
R2-ISS分期及重新分期的R2-ISS分期患者的总生存（A）和无进展生存（B）曲线

## 讨论

研究显示MM的高度异质性是由其生物学特性所决定的，特别是细胞遗传学的改变，包括染色体数目和结构异常、基因表观遗传学异常和细胞信号通路紊乱等[6]–[7]，与生存密切相关，是目前MM危险分层的重要依据。随着MM用药指导及改善预后的需求，近10年MM预后相关的危险分层在不断地改进优化。2015年IMWG在ISS分期的基础上引入t（4；14）、t（14；16）和del（17p），提出了R-ISS分期[3]。然而，该分期存在着一定局限，约60％的MM患者被划入Ⅱ期，其中包括具有不同进展或死亡危险的患者，因此需要提出更准确、可靠的分期系统来优化MM的预后分层。2018年更新的mSMART3.0危险分层高危组中纳入了1q+，同时提出了双/三打击的概念，并指出具有高危特征的双/三打击患者需要更强的治疗方案[8]–[10]。2022年EMN基于R-ISS分期纳入了不良预后因素1q+，提出了新的危险分层系统R2-ISS，该研究通过对每个单一基线危险因素给予评分值，以累加的方式确定了4个分离良好且独立的预后组，比较分析各组的OS和PFS，结果显示R2-ISS分期可以较好区分不同预后的MM患者，特别是R-ISS Ⅱ期中生存结果较差的群体[2]。值得注意的是，R2-ISS分期是基于大量临床试验数据开发并验证的，需要在真实世界中不断验证并改进。同时关于1q+的赋值仍存在争议。

本研究根据R2-ISS分期对本中心326例患者的临床数据进行分层，得到4个预后不同的分组，不同组间OS和PFS期差异均有统计学意义。不同的是，本研究队列在年龄、ISS分期、R-ISS 分期、1q+、一线治疗方案、auto-HSCT比例与EMN队列的可评估训练集分布上差异有统计学意义，Chen等[11]研究队列同样存在类似差异，表明即使差异存在R2-ISS仍然保持着良好的预后能力。在EMN研究中R2-ISS分期被验证可以更好地区分R-ISS Ⅱ期中不良预后的患者，因此，本研究根据R2-ISS分期系统将R-ISS Ⅱ期患者重新分层，不同分期中位PFS和OS期差异均具有统计学意义（*P*<0.001），表明R-ISS Ⅱ期患者在生存率方面存在高度异质性，可以通过R2-ISS分期进行精准分层。与EMN的研究对象分布不同的是，本研究中Ⅲ～Ⅳ期占比大于EMN研究中的50％，与国内区域流行病学调查研究结果类似[12]。

与R-ISS相比，R2-ISS分期增加了1q+这一指标[2]。1q+是MM最常见的细胞遗传学异常之一，包括gain（1q）（3个总拷贝）和amp（1q）（≥4个总拷贝），约40％的MM初诊患者会发生1q+[13]。然而，关于1q+在MM中的预后影响及在R2-ISS分期中的赋值仍存在争议。这主要是由于不同实验室细胞遗传学的报告和注释缺乏一致性，特别是在报告1号染色体上的异常时，相当一部分实验室报告的注释没有注明1q拷贝数[14]。一项meta分析显示gain（1q）与高危临床特征、高危疾病和不良生存相关，但在对其他高危细胞遗传学异常进行校正后，未发现1q+是独立的预后因素[15]–[16]，而amp（1q）患者无论有无其他细胞遗传学异常，其生存率均较差[17]。Abdallah等[18]的临床研究发现，在1q+患者中，无论1q拷贝数或其他细胞遗传学异常，以及患者是否在诱导治疗中接受了PI、IMiD或这两种治疗，患者OS均降低。总而言之，有1号染色体异常［包括1q+或del（1p）］患者的结局劣于无异常患者[19]–[20]。和EMN相同，本研究将gain（1q）和amp（1q）合并赋值，分析发现伴1q+患者的中位PFS期和OS期更短，从而得出1q+是影响NDMM患者预后的独立危险因素，这也与EMN研究结论一致。由于技术限制，关于gain（1q）和amp（1q）患者的预后分析比较有待进一步研究。Schmidt等[5]的研究指出，1q+与多发性骨髓瘤的早期进展相关，gain（1q）合并t（4；14）或del（17p）患者的PFS较差，这提示存在“双打击”效应。本中心数据显示1q+检出率高于EMN队列的可评估训练集，与我国NDMM人群1q+发生率较高事实相符[21]，因此检出含1q+在内的双打击概率相应增大。本研究结果显示含1q+在内的双打击患者中位OS期和PFS期更短。尽管PFS无统计学意义，考虑可能是样本量偏小所致。将双打击中1q+赋值调整至1，重新分组后的四组中位PFS和OS差异仍有统计学意义，而Ⅲ期患者比例下降，且无论是否存在1q+预后均无差异。因此，本研究认为合并1q+在内的“双打击”遗传学异常患者预后更差，应尽早采用新药在内的多药联合方案±移植治疗以延长生存时间。有关1q+的赋值及预后需要更大样本量的多中心临床研究来证实。这种对危险因素赋值后累加评分的方法赋予R2-ISS分期更灵活的性质，更容易进行调整并纳入其他新的危险因素，比如循环浆细胞、髓外病灶、TP53突变等。2022年Mayo诊所提出了一个五因素三层赋值评分的MASS分期[22]，包括高危险IgH易位、1q+、del（17p）、ISS Ⅲ期和LDH升高，与R2-ISS不同的是，加入了mSMART3.0危险分层提出的双/三打击的概念，每个危险因素均赋值为1，这两种分期系统的优势比较需要后续进一步研究。因本研究样本量有限，针对已有的高危因素后期仍需进一步随访并验证。

综上，R2-ISS分期是一种简单实用的分期系统，具有良好的兼容性，指标易于获得，能够精准分层R-ISS分期Ⅱ期患者，从而更好地应用于临床实践指导治疗。含1q+在内的“双打击”患者预后较差，其赋值的调整为后续R2-ISS分期的进一步优化提供了新的思路。

## References

[1] Palumbo A, Anderson K (2011). Multiple myeloma[J]. N Engl J Med.

[2] D'Agostino M, Cairns DA, Lahuerta JJ (2022). Second Revision of the International Staging System (R2-ISS) for Overall Survival in Multiple Myeloma: A European Myeloma Network (EMN) Report Within the HARMONY Project[J]. J Clin Oncol.

[3] Palumbo A, Avet-Loiseau H, Oliva S (2015). Revised International Staging System for Multiple Myeloma: A Report From International Myeloma Working Group[J]. J Clin Oncol.

[4] Ross FM, Avet-Loiseau H, Ameye G (2012). Report from the European Myeloma Network on interphase FISH in multiple myeloma and related disorders[J]. Haematologica.

[5] Schmidt TM, Barwick BG, Joseph N (2019). Gain of Chromosome 1q is associated with early progression in multiple myeloma patients treated with lenalidomide, bortezomib, and dexamethasone[J]. Blood Cancer J.

[6] Morgan GJ, Walker BA, Davies FE (2012). The genetic architecture of multiple myeloma[J]. Nat Rev Cancer.

[7] Munshi NC, Anderson KC, Bergsagel PL (2011). Consensus recommendations for risk stratification in multiple myeloma: report of the International Myeloma Workshop Consensus Panel 2[J]. Blood.

[8] Dispenzieri A, Rajkumar SV, Gertz MA (2007). Treatment of newly diagnosed multiple myeloma based on Mayo Stratification of Myeloma and Risk-adapted Therapy (mSMART): consensus statement[J]. Mayo Clin Proc.

[9] Kumar SK, Mikhael JR, Buadi FK (2009). Management of newly diagnosed symptomatic multiple myeloma: updated Mayo Stratification of Myeloma and Risk-Adapted Therapy (mSMART) consensus guidelines[J]. Mayo Clin Proc.

[10] Mikhael JR, Dingli D, Roy V (2013). Management of newly diagnosed symptomatic multiple myeloma: updated Mayo Stratification of Myeloma and Risk-Adapted Therapy (mSMART) consensus guidelines 2013[J]. Mayo Clin Proc.

[11] Chen H, Zhou N, Hu X (2023). The applicability of the Second Revision of the International Staging System for patients with multiple myeloma receiving immunomodulatory drugs or proteasome inhibitor-based regimens as induction treatment: A real-world analysis[J]. Hematol Oncol.

[12] 王 得印, 郝 云良, 赵 同峰 (2017). 济宁市多发性骨髓瘤流行病学调查研究[J]. 国际输血及血液学杂志.

[13] Schmidt TM, Fonseca R, Usmani SZ (2021). Chromosome 1q21 abnormalities in multiple myeloma[J]. Blood Cancer J.

[14] Yu Y, Brown Wade N, Hwang AE (2020). Variability in Cytogenetic Testing for Multiple Myeloma: A Comprehensive Analysis From Across the United States[J]. JCO Oncol Pract.

[15] Fonseca R, Van Wier SA, Chng WJ (2006). Prognostic value of chromosome 1q21 gain by fluorescent in situ hybridization and increase CKS1B expression in myeloma[J]. Leukemia.

[16] Neben K, Jauch A, Bertsch U (2010). Combining information regarding chromosomal aberrations t(4;14) and del(17p13) with the International Staging System classification allows stratification of myeloma patients undergoing autologous stem cell transplantation[J]. Haematologica.

[17] D'Agostino M, Ruggeri M, Aquino S (2020). Impact of gain and amplifification of 1q in newly diagnosed multiple myeloma patients receiving carfifilzomib-based treatment in the forte trial[J]. Blood.

[18] Abdallah N, Greipp P, Kapoor P (2020). Clinical characteristics and treatment outcomes of newly diagnosed multiple myeloma with chromosome 1q abnormalities[J]. Blood Adv.

[19] Giri S, Huntington SF, Wang R (2020). Chromosome 1 abnormalities and survival of patients with multiple myeloma in the era of novel agents[J]. Blood Adv.

[20] Varma A, Sui D, Milton DR (2020). Outcome of Multiple Myeloma with Chromosome 1q Gain and 1p Deletion after Autologous Hematopoietic Stem Cell Transplantation: Propensity Score Matched Analysis[J]. Biol Blood Marrow Transplant.

[21] You H, Jin S, Wu C (2022). The independent adverse prognostic significance of 1q21 gain/amplification in newly diagnosed multiple myeloma patients[J]. Front Oncol.

[22] Abdallah NH, Binder M, Rajkumar SV (2022). A simple additive staging system for newly diagnosed multiple myeloma[J]. Blood Cancer J.

